# A New Optical Surface Measurement Method with Iterative Sparsity-Constrained Threshold Phase Retrieval Algorithm

**DOI:** 10.1155/2014/548395

**Published:** 2014-04-09

**Authors:** Yi Niu, Yang Liu, Guangming Shi, Dahua Gao, Guo Li

**Affiliations:** ^1^School of Electronic Engineering, Xidian University, Xi'an 710071, China; ^2^Department of Electrical and Computer Engineering, McMaster University, Hamilton, ON, Canada L8S 4L8; ^3^School of Science, Air Force Engineering University, Xi'an 710051, China

## Abstract

Due to its low complexity and acceptable accuracy, phase retrieval technique has been proposed as an alternative to solve the classic optical surface measurement task. However, to capture the overall wave field, phase retrieval based optical surface measurement (PROSM) system has to moderate the CCD position during the multiple-sampling procedure. The mechanical modules of CCD movement may bring about unexpectable deviation to the final results. To overcome this drawback, we propose a new PROSM method based on spatial light modulator (SLM). The mechanical CCD movement can be replaced by an electrical moderation of SLM patterns; thus the deviation can be significantly suppressed in the new PROSM method. In addition, to further improve the performance, we propose a new iterative threshold phase retrieval algorithm with sparsity-constraint to effectively reconstruct the phase of wave field. Experimental results show that the new method provides a more simple and robust solution for the optical surface measurement than the traditional techniques and achieves higher accuracy.

## 1. Introduction

The accuracy of optical surface is critical to the imaging system such as medical imaging, space telescope, and microscopic imaging. Therefore, a comprehensive test should be accomplished after the manufacture of every optical surface. Recently, phase retrieval based optical surface measurement (PROSM) technique attracts extensive attention due to its simple measurement model, acceptable accuracy, and diversity of test objects. Inspired by Fourier optics theory [1, 2], PROSM was first proposed by Fienup group in 2004 [3–5]. And the paper [6] demonstrates the transverse translation-diverse phase retrieval method in the situations where detected intensity patterns are undersampled. In paper [7], an improved phase retrieval algorithm is proposed to overcome the measurement and calculation difficulty for aspheric surface.

The work principle of PROSM is to reconstruct the phase information of the wave filed from the intensity measurements. This procedure is mathematically a typical inverse problem. Solving this problem requires sufficient observations that multiple sampling should be conducted. To accomplish this, traditional PROSM system has to moderate the CCD position after every sampling; thus mechanical control of CCD is inevitable. The mechanical modules of CCD movement may bring about unexpectable deviation to the final results. To overcome this drawback, we investigate a new solution to accomplish multiple sampling with fixed position of the CCD.

The intrinsic reason of moderating CCD position is to observe multiple intensity measurements in different phases. In other words, the CCD movement equals a modulation of the phase of wave filed. Instead of modulating the phase mechanically, we propose a new solution to modulate the phase electronically. Recently, a novel optical device named spatial light modulator (SLM) has been widely adopted in optical system such as optical computing [8–10] and holographic display technique [11–13]. SLM is an electrical device that imposes some form of spatially varying modulation on the light beam that both the intensity and the phase can be adjusted.

We propose a novel electrical PROSM solution based on SLM. All of the equipment in our system is placed in 4f setup without any mechanism module. The moderation of CCD position is accomplished by adjusting the phase of the light beam passing through the SLM. Therefore, multiple intensities can be captured at the image plane of 4f optical setup without any movement of CCD.

The remaining task is how to retrieve the phase from the captured intensities. As we related before, phase retrieval is an ill-pose inverse problem and has multiple solutions [14–16]. In pursuit of the optimum solution, appropriate prior knowledge is required. This work takes advantages of the sparsity prior of the wave front.

Sparse approximations are a widely adopted regularization technique in many image processing techniques [17–19]. Phase retrieval with sparse constraint has been put forward by Donoho and Elad in 2003 [20]. The author assumes that there exists a basis consisting of a small number of items where the phase of the wave field can be represented exactly or approximately with high accuracy. Based on this assumption, the author proposes a 4f-setup based optical system that the phase can be retrieved effectively via a 4f Sparse Phase Amplitude Reconstruction (4f-SPAR) algorithm.

An ad hoc solution for the optical surface measurement task is to combine the 4f-SPAR algorithm with PROSM system directly. However, the 4f-SPAR algorithm cannot fully explore the sparsity prior of optical surfaces. To be specific, the retrieval performance of 4f-SPAR algorithm strongly relies on the accuracy of sparsity models (the trained dictionary), but the sparsity model estimation needs the very original data. To solve this chicken-and-egg dilemma, 4f-SPAR separates the phase retrieval task into two steps. The first step is to get an initial estimation of the phase data via traditional GS algorithm. Then, reconstructed phase data is refined via a sparsity model based phase retrieval technique in the second step. Therefore, the GS based initial estimation plays an important role in the 4f-SPAR phase retrieval algorithm.

In traditional GS method, the phase of the wave field is reconstructed by projecting the amplitude of the wave field into time domain and Fourier domain iteratively. A strong prior knowledge is neglected by the GS method. In fact, almost all the optical surfaces are smooth, and it can be assumed that the phase signals are sparse in frequency domain. Relying on this assumption, we propose a novel iterative threshold phase retrieval algorithm with sparsity-constraint (ITPRS) to further improve the phase retrieval performance. Like the widely used compressive sensing techniques, *L*_1_ norm is adopted to approximate the *L*_0_ norm and characterize the sparsity of signals. ITPRS follows the same iteration steps as the GS method, but suppressing the *L*_1_ norm of the DCT coefficients during the iteration.

Another advantage of the proposed ITPRS is that the amplitude information of the wave filed is available. To be specific, the amplitude of emitted light is set to a constant level in the surface measurement process. Therefore, only the phase information should be considered in the restoration of wave field. The ITPRS technique takes advantage of the reduction of variables in both performance and complexity.

The rest of this paper is organized as follows. In Section 2, the general system for optical surface measurement is described. The basic principle and model for ITPRS are presented in Section 3. Experimental results are reported and discussed in Section 4.  Section 5 is the conclusion of this paper.

## 2. The System for Optical Surface Measurement

**Figure 1:**
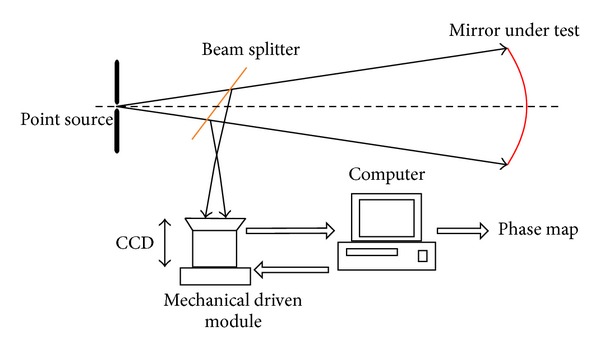
Framework of traditional PROSM technique.

The framework of traditional PROSM technique is shown in Figure 1. The under test mirror is illuminated by a point source and the reflected light is collected by a CCD camera passing through a beam splitter. The computer controls the mechanical module to change the position of CCD around the focus plane. The intensities of wave filed are captured and stored after every CCD movement.

The mechanical adjusting of CCD position may bring about unexpectable deviation to the final results. To overcome this drawback, we investigate the possibility of replacing the mechanical module by electrical devices. Reminding the principle of Fourier optics theory, the CCD movement is equivalent to the adjusting of phase of wave field. The work of Candès [16] and Donoho and Elad [20] shows that the phase modulation task can be accomplished by a new electrical device named SLM. Inspired by their work, we investigate the possibility of using the electrical device SLM to replace the mechanical CCD control module.

**Figure 2:**
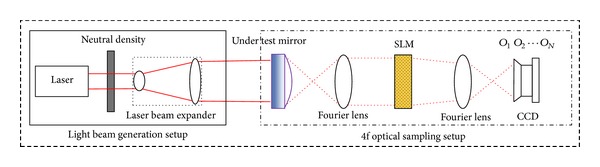
Framework of data sampling module used for optical surface measurement.

The novel electrical PROSM system is illustrated in Figure 2; the system consists of two parts: light beam generation setup and date sampling setup. The light beam generation setup is used for producing the desired beam for the optical surface measurement, and the function of the data sampling setup is to gather enough available information of the phase of wave field.

To get the ideal sampling result, the light beam which projects onto the under test mirror should be uniform and the intensity should be constrained in an appropriate level to prevent the CCD from saturating. Therefore, we use laser to generate the illumination beam and adopt a neutral density to control the light intensity. The uniform laser beam is then expanded by a beam expander to cover the whole surface.

**Figure 3:**
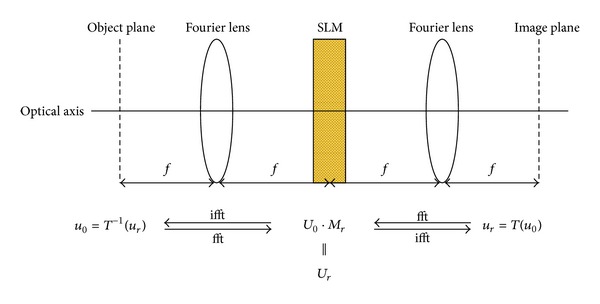
The 4f optical measure setup and brief GS based computational model.

The data sampling setup consists of 4 types of equipment, a SLM, a CCD, and two identical Fourier lens with focal length *f*. These types of equipment are placed in order as shown in Figure 2, and the distance between each other is *f*. The under test mirror and the equipment of the data sampling setup form a classical 4f optical setup. The under test mirror and the CCD are located at the object plane and image plane of the 4f-setup respectively, as shown in Figure 3.

The uniform light wave passes through the under test mirror and can be modulated by the SLM then collected by the CCD. For a better description of this process, the light beam in Figure 2 is denoted by dash lines to represent that actual propagation of light is related to both the features of the under test mirror and the SLM modulation. SLM adjusts the phase of the wave field randomly before every CCD shut.

Repeating this process *N* times, multiple intensity data (*O*_1_, *O*_2_,…, *O*_*N*_) is acquired, which carries sufficient information of wave field. As we discussed above, the new PROSM system is composed of optical and electrical devices. Therefore, the unexpectable deviation caused by the traditional mechanical module can be significantly suppressed. The remaining task is how to retrieve the phase of the wave field and deduce the parameters of the under test mirror.

## 3. Basic Principle and Model of Iterative Threshold Phase Retrieval with Sparsity-Constraint 

In pursuit of higher phase retrieval performance, we propose an iterative threshold phase retrieval algorithm with sparsity-constraint (ITPRS) to reconstruct the phase from the multiple intensity sampling data. As the traditional PROSM technique, we also assumed that the wave front is coherent that Fresnel paraxial approximation can be used to model the optical surface measurement procedure.

The main contribution of ITPRS is that we exploit strong prior information of the optical surface which has always been ignored by the previous phase retrieval algorithms. In fact, almost all the optical surfaces are smooth; thus the wave field can be considered as a sparse signal in frequency domain. As we will discuss later, the sparsity prior works as an essential regulation term in the ITPRS technique and brings significant gain to the phase retrieval performance.

Another advantage of the ITPRS is that the amplitude information of the wave filed is available in the novel electrical PROSM system. The amplitude of emitted light is set to a constant level (*ℑ*) in the process of the measurement. Since the amplitude information is available, we only need to reconstruct the phase information of the wave field. The reduction of variables can significantly improve the phase retrieval performance and also reduce the complexity.

To facilitate the introduction of the ITPRS technique, we use *u*_0_(*x*) and *u*_*r*_(*x*) to denote the wave front of the object plane and the image plane of the 4f optical setup, respectively, as shown in Figure 3. The coordinate of the object and image plane is represented as *x* = (*x*_1_, *x*_2_), and the coordinate of the Fourier plane (SLM) is denoted by *v* = (*v*_1_, *v*_2_).

We first define two operators *F* and *F* ^−1^ to represent the Fourier and inverse Fourier transform as follows:

(1) $$\begin{array}{c} h\left(v\right)=F\left\{u\left(x\right)\right\}\left(v\right)=\iint_{R^{2}}u\left(x\right)\exp\left(-2\pi j\left(v,x\right)\right)dx_{1}dx_{2},\\ u\left(x\right)=F^{-1}\left\{h\left(v\right)\right\}\left(x\right)=\iint_{R^{2}}h\left(v\right)\exp\left(2\pi j\left(x,v\right)\right)dv_{1}dv_{2}. \end{array}$$

According to the Fourier optics theory [2, 20], the wave field in the focal plane of the first lens (SLM) is as follows:

(2) $$\begin{array}{c} U_{0}\left(v\right)=\frac{1}{j\lambda f}F\left\{u_{0}\left(x\right)\right\}\left(\frac{v}{\lambda f}\right), \end{array}$$

where *λ* is the wave length of light and *f* is the focal length. Defining the phase modulation function of SLM by *M*_*r*_, the wave filed in the image plane (CCD) can be written as

(3) $$\begin{array}{c} u_{r}\left(x\right)=\frac{1}{j\lambda f}F^{-1}\left\{U_{0}\cdot M_{r}\right\}\left(\frac{x}{\lambda f}\right). \end{array}$$

Combining (2) and (3), we can obtain the transfer function of the 4f optical surface as

(4) ur(x)=1jλfF−1{U0·Mr}(xλf)=1jλfF−1{1jλfF{u0(x)}(vλf)·Mr}(xλf).

For simplicity reasons, we rewrite (4) by

(5) $$\begin{array}{c} u_{r}\left(x\right)=T\left(u_{0}\left(x\right),M_{r}\right),\\ u_{0}\left(x\right)=T^{-1}\left(u_{r}\left(x\right),M_{r}\right), \end{array}$$

where *T* ^−1^(*u*_*r*_(*x*), *M*_*r*_) is the inverse transfer function from *u*_*r*_(*x*) to *u*_0_(*x*).

Reminding that the optical surface can be regarded as a sparse signal in frequency domain, we define the objective function of ITPRS algorithm as follows:

(6) u0(x)=arg⁡min⁡u0(x),αφ ∑r=1N||Or−|T(u0(x),Mr)|2||2+λφ·||αφ||1s.t. abs(u0)=sqrt(ℑ), abs(ur)=sqrt(Or),

where the first term is the fidelity term, the second term is the regularization term, and *α*_*ϕ*_ is the coefficients of *u*_0_ at some sparse domain; here we use discrete cosine transform; thus *α*_*ϕ*_ = DCT(*u*_0_), and *λ*_*φ*_ is a weight parameter. abs(·) and angle(·) denote the amplitude (modulus) and phase (angle), respectively.

The objective function of (6) is a classical *L*_1_ and *L*_2_ norm optimization problem which can be easily solved by techniques such as convex relaxation method [21] and iterative thresholding method [22]. In this paper, we adopt the iterative thresholding method.

Due to the sparsity prior, the solution of (6) provides a much better estimation of the very original wave field than traditional GS technique. However, in pursuit of higher retrieval performance and robustness, postprocessing technique is adopted to further exploit the sparsity prior, especially the nonlocal sparsity of the wave field.

BM3D method [22] has been proofed as an efficient nonlocal based postprocess technique for the phase retrieval task [20]. Therefore, we only adopted the solution of (6) as a coarse grain estimation of the wave filed, then used BM3D technique to refine it. Solving (6) and the BM3D postprocessing are two key techniques of the ITPRS technique.

The details of the proposed ITPRS algorithm are shown in Algorithm 1.

Given the final retrieved phase *u*_0_, the under test optical surface can be easily derived by

(7) $$\begin{array}{c} \varphi =\frac{2\pi }{\lambda }n\cdot \Delta x, \end{array}$$

where *n* is refractive index of light and Δ*x* is the data of optical surface.

## 4. Experimental Results

Simulation experiments are conducted to evaluate the performance of our method.

In our experiments, the under test surface is assumed to be cosine distributed and the phase of the surface is denoted by *w*. We conduct 10 samples to reconstruct the phase.

The amplitude of the wave front *ℑ* is set to 1 (pixel value, ranging from 0 to 1). We use a random value *z* to represent the initial phase of wave field and the phase after the optical surface is *Z* = *z* + *w*. Thus, *u*_0_ can be written as *u*_0_ = *ℑ* · exp⁡(*j* · *Z*), and the multiple intensity data *O*_*r*_ can be derived by ||*u*_*r*_||^2^. For a faithful simulation of the real environment, a Gaussian noise *ε* is added to *O*_*r*_. *ε* is with zero mean and the variance *σ*_*r*_ is equal to 0.05.

**Figure 4:**
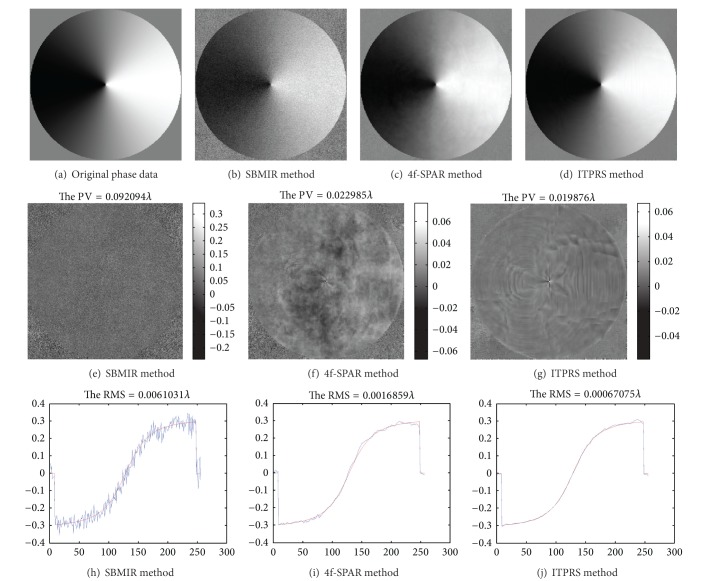
Results of phase retrieval in different methods: the first row (a) is the original phase data and (b)–(d) are the reconstructed phase of wave field; the second row (e)–(g) is the residuals between the original phase data (a) and the reconstructed phase data; the last row (h)–(j) is a random line of phase data between the reconstructed (blue) and original (red) phase data.

We compare the phase retrieval performance of ITPRS with two classical techniques SBMIR [15] and 4f-SPAR [20]. The results are illustrated in Figure 4. The first row of Figure 4 represents the overall visual comparison of the retrieved phases. In the second row of Figure 4, we compare the residuals of each technique, and the last row is the comparison of a random line of the phase matrix. It can be observed that ITPRS provides a much more smooth and faithful estimation of the original phase than the other two techniques, due to the fine exploitation of the sparsity prior.

The final surface measurement results are reported in Table 1. We use two commonly used objective metric (peak value) PV and (root-mean square) RMS to evaluate the objective performances of the three techniques. It is not surprising to observe that, due to a more faithful retrieval of the phases, the proposed ITPRS technique significantly improves the objective performance than the two competitors. To be specific, the PV and RMS performance are improved by 13.4% and 60%, respectively, than 4f-SPAR. This is quite remarkable, since the 4f-SPAR technique reports the best phase retrieval performance in literature.

**Figure 5:**
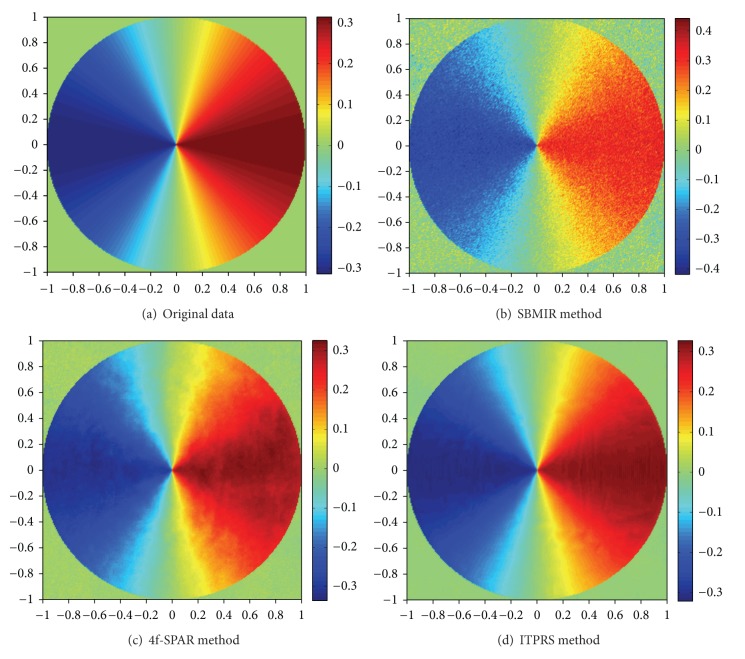
Color results of the reconstructed surface.

The visual comparison of the reconstructed surface is illustrated in Figure 5. It can be observed that the proposed ITPRS technique provides more clear and sharp boundaries than the other two techniques. Another observation is that, due to the cooperative work of sparsity based phase retrieval and BM3D based postprocessing, the noises are significantly suppressed in the our system.

## 5. Conclusions

We proposed a new optical surface measurement solution with iterative sparsity constrained threshold phase retrieval algorithm. Comparing with the traditional PROSM system, the mechanical CCD movement module can be replaced by electrical device SLM. In addition, we incorporate the sparsity prior into the traditional phase retrieval techniques which provides a more efficient way to faithfully reconstruct the phase of the under test surfaces. Simulation experiments show that the proposed system and phase retrieval algorithm achieved significant improvement than the traditional techniques. The proposed method provides a potential way to improve the current industry of optical manufacturing and measurement.

## Figures and Tables

**Algorithm 1:**
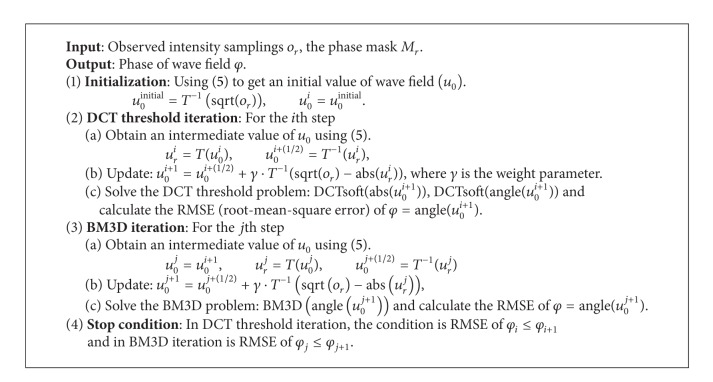
ITPRS.

**Table 1:** The PV and RMS of different techniques.

	SBMIR	4f-SPAR	ITPRS
PV	0.092094	0.022985	**0.019876** **(13.4% less)**

RMS	0.0061031	0.0016859	**0.00067075** **(60% less)**
